# Multimodal Practice for Mobilizing Response: The Case of Turn-Final *Tu Vois* ‘You See’ in French Talk-in-Interaction

**DOI:** 10.3389/fpsyg.2021.659340

**Published:** 2021-10-22

**Authors:** Ioana-Maria Stoenica, Sophia Fiedler

**Affiliations:** ^1^Center for Applied Linguistics, University of Neuchâtel, Neuchâtel, Switzerland; ^2^Institut für Germanistik, University of Hamburg, Hamburg, Germany

**Keywords:** *tu vois*, you see, response mobilization, gaze conduct, multimodality, affiliation, alignment, preference

## Abstract

One of the most frequent verbal expressions that people use when interacting with each other in French is *tu vois* ‘you see’ (Cappeau, 2004). Drawing on interactional linguistics and multimodal analysis, we examine the interactional functioning of this verbal expression when occurring in turn-final position. Previous studies on *tu vois* ‘you see’ in this position document only its use for marking the end of an utterance or for turn-yielding. The following aspects have thus far remained unexplored: The interactional environment in which the construction occurs, how it is connected to the speaker’s embodied conduct, the way in which it contributes to mobilizing a response from the recipient, as well as the nature of this response. Our paper addresses these issues and shows that turn-final *tu vois* ‘you see’ is systematically produced with a final rising intonation and coupled with the speaker’s gaze directed to the recipient. This multimodal practice is recurrently deployed in turns conveying the speaker’s emotional stance, in turns performing a dispreferred action, like disagreeing, and in turns claiming insufficient knowledge. The response that is invited using this multimodal practice is distinctly tailored to each of these actions: an affiliative response, an aligning response, and a response addressing the prior speaker’s claim of insufficient knowledge from the recipient’s own point of view. By presenting an in-depth study of the action sequences in which *tu vois* ‘you see’ is employed, as well as of its multimodal packaging, this contribution highlights the prospective, i.e., response-mobilizing potential of this interactional resource and shows that its use entails sequential implications even when it accompanies actions that project only weakly a response from the recipient.

## Introduction

One of the most frequent verbal expressions that people use when interacting with each other in French is *tu vois* ‘you see’ (Cappeau, 2004). The use of this linguistic resource is highly recurrent in spoken interaction, where it is involved in the construction of turns and in the management of turn-taking and turn-allocation. As such, this expression is of a functional-pragmatic nature and cannot be adequately described in morpho-syntactic terms (Mosegaard Hansen, 1998) but rather in relation to the interactional functions that it locally fulfills in the conversational architecture.

Drawing on interactional linguistics (Ochs et al., 1996; Hakulinen and Selting, 2005; Couper-Kuhlen and Selting, 2018) and on multimodality (Goodwin, 1979; Deppermann, 2013a, b; Streeck, 2013; Kärkkäinen and Thompson, 2018; Keevallik, 2018; De Stefani, 2019; Pekarek Doehler, 2019), we set out to explore in this paper the conversational use of *tu vois* ‘you see’ in French talk-in-interaction. More specifically, we aim to examine the interactional functioning of this construction in turn-final position, as briefly illustrated by excerpt (1) from our data:


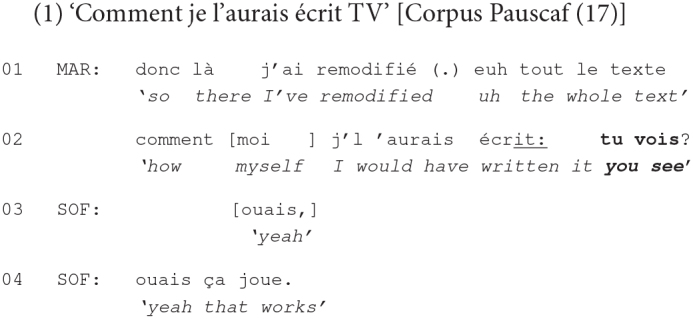


The topic of this study has emerged naturally from the conversational data that we examined. Following Cappeau (2004), we identified all recurrent linguistic structures combining the second-person singular pronoun and a verb in the present tense without a complement (clause) and found that *tu vois* ‘you see’ was the most frequent expression used in our data. It also appeared to be most recurrently used in turn-final position (see section “Data and Multimodal Features of Turn-Final *Tu Vois* ‘You See”’ *infra*). Previous studies on *tu vois* ‘you see’ in this position document only its use for marking the end of an utterance or for turn yielding by signaling the exit from the turn (see section “Background” *infra*). Complementing this research, our paper aims to provide a detailed account of the use of this construction in turn-final position, by exploring the conversational actions and also the participants’ systematic embodied conduct deployed in this sequential environment – something that has largely remained uninvestigated in the existing literature.

This contribution intends hence to show that *tu vois* ‘you see’ forms a multimodal practice featuring a consistent prosodic and embodied pattern that is deployed in turns conveying the speaker’s emotional stance, performing a dispreferred action, or claiming insufficient knowledge. Even when these turns project only weakly some action on the coparticipants’ part (Stivers and Rossano, 2010; Auer, 2017, 2021), as, for example, the turns claiming insufficient knowledge, they still appear to overwhelmingly receive a relevant response from recipients (see section “Data and Multimodal Features of Turn-Final *Tu Vois* ‘You See”’ *infra*). The analyses will show that it is the use of *tu vois* ‘you see’ with final rising intonation, in correlation with the speaker’s gaze addressed to the coparticipant, that contributes to mobilizing a response from the latter. Moreover, it will be shown that this multimodal practice is dynamically deployed, triggering a distinct response from the recipient in the three action sequences mentioned above.

The remainder of the paper is organized as follows: Section “Background” outlines the background of our study, reviewing some of the works that have dealt with the specific construction that we are interested in. Section “Data and Multimodal Features of Turn-Final *Tu Vois* ‘You See”’ describes the multimodal features of *tu vois* ‘you see’ and the activities performed in the sequential environment of its use, impacting on its production and interpretation. Section “Interactional Workings of Turn-Final *Tu Vois* ‘You See”’ presents multimodal analyses showing the speakers’ use of turn-final *tu vois* ‘you see’ in different action sequences. Section “Discussion and Conclusion” summarizes our main findings and discusses their implications for the understanding of the conversational use of *tu vois* ‘you see’ as a multimodal practice involved in the organization of turns and actions.

## Background

The construction *tu vois* ‘you see’ belongs to a type of verbal expression that has been discussed in the literature under various names. According to Bolly (2010), in French, these expressions are termed: *marqueurs discursifs propositionnels* (Andersen, 1997, 2007), *énoncés parenthétiques* (Debaisieux, 2008), *constructions parenthétiques* (Bolly, 2012), *constructions à verbe recteur faible* (Blanche-Benveniste and Willems, 2007), while in English they are labeled as: *(reduced) parenthetical clauses* (Schneider, 2007), *epistemic parentheticals* (Dehé and Wichmann, 2010) or *comment clauses* (Brinton, 2008).

Heavily employed in spoken interaction, *tu vois* ‘you see’ has been documented to hold several features in common with discourse markers^1^ : It is morphologically invariable, syntactically optional, it has a relatively free position within the utterance in which it occurs, the truth-value of which it does not modify, and it conveys a subjective and intersubjective meaning, establishing thus “shared understanding between social actors” (Raymond, 2019: 182; see also Andersen, 2007; Bolly, 2010; Traugott, 2010). This discourse marker-like use of *tu vois* ‘you see’ is in line with previous work on complement-taking predicates involving mental verbs (such as *I mean*, *I think*, and *I guess*). These verbs have been documented to lose, in some of their conversational uses, their syntactic and semantic status as main clauses and become markers of epistemic stance, while still keeping traces of their original semantics (see the seminal paper of Thompson and Mulac, 1991 and Kärkkäinen, 2003 for English; Laury and Okamoto, 2011 for Japanese; Pekarek Doehler, 2011 for French; Maschler, 2012 for Hebrew). Similar findings have been also attested for some of the interactional uses of *I don’t know* (see Keevallik, 2016 for Estonian; Helmer et al., 2016 for German; Lindström and Karlsson, 2016 for Swedish; Pekarek Doehler, 2016 for French).

Research on verbal expressions that are similar to *tu vois* ‘you see’, such as the construction ‘you know,’ is also relevant for our study (see Jefferson, 1972 for English; Keevallik, 2003 for Estonian; Lindström and Wide, 2005 for Swedish; Asmuß, 2011 for Danish). Jefferson (1972), for example, investigating tag-positioned address terms in closing sequences, has shown that in turn-final position ‘you know’ indicates the completion of the turn and thus possible turn-transition. Additionally, it may also serve to avoid a pause between a prior problematic component and the recipient’s response. Asmuß (2011) has examined the use of ‘you know’ (*du ved* in Danish) essentially in turn-initial and mid-turn positions, with only one example in turn-final position, as a resource for pursuing agreement, by appealing to shared knowledge, in an environment of potential disagreement. Recently, Clayman and Raymond (2021) have argued that English ‘you know’ functions as an alignment token, “one that *invokes a convergent orientation between recipient and speaker*” (*ibid*.: 2, original emphasis). They identify two different subgroups for this function: Alignment that allows the recipient to show correct understanding of what the speaker said (‘intersubjective alignment’) and alignment that affiliates with the speaker’s stance (‘affiliative alignment’). One important feature of both groups is the environment in which ‘you know’ is used: when affiliation or/and understanding emerge as ‘non-given’ or even problematic in the ongoing -sequence. Interestingly, some of our findings, namely those concerned with the use of *tu vois* ‘you see’ in turns conveying the speaker’s emotional stance, converge with Clayman and Raymond (2021) results on the use of ‘you know’ for achieving ‘affiliative alignment.’

Particularly germane to our analytic focus are works that have specifically examined the use of *tu vois* ‘you see’ in French data. Bolly (2010, 2012), for instance, has studied the pragmaticalization^2^ (Erman and Kotsinas, 1993; Dostie, 2004) of *tu vois* ‘you see’ across the centuries, from pre-classical to contemporary French. Using both written and oral data, Bolly shows that the semantics of *tu vois* ‘you see’ has evolved throughout centuries from a meaning based on visual perception to pragmatic uses (with what she calls *interpersonal functions*), passing through cognition-related usages (i.e., connected to the process of understanding). The author has also shown that this semantic movement of *tu vois* ‘you see’ is coupled with a syntactic one, this construction evolving from complex syntax to syntactically autonomous usages. Bolly has also stated that the pragmaticalization of *tu vois* ‘you see’ is still in-progress nowadays, the pragmatic uses co-existing with the more ancient uses of this verbal expression, based on visual perception.

Andersen (1997, 2007) has studied the functions of *tu vois* ‘you see’ in spoken French according to the position in which it occurs in the utterance. In anteposition, *tu vois* ‘you see’ has been found to mark the introduction of a new piece of information, while in postposition, it has been documented to indicate the end of an utterance (functioning similarly to what Vincent (1993) called in French *ponctuants*). In a parallel line of research, Mondada (2004) and Détrie (2010) have both examined *tu vois* ‘you see’ in French conversations and have documented that, in turn-initial position, it serves to reactivate the coparticipant’s attention, and in turn-final position, it signals the speaker’s exit from the turn.

These previous studies have been concerned with the use of *tu vois* ‘you see’ in different positions in the turn. While this approach provides a nice picture of the general functioning of this expression, it remains essentially grounded on the macro-level of the conversation. This means that the identified functions of *tu vois* ‘you see’ are not minutely related to the specific conversational actions performed in the respective sequential positions. Moreover, the functions that have been documented for *tu vois* ‘you see’ in turn-final position are of fundamentally retrospective nature. The way in which this construction contributes to mobilizing a response from the recipient, as well as the nature of this response have thus far remained unexplored.

Our paper addresses these issues, by offering a detailed account of the conversational actions performed in the turns to which *tu vois* ‘you see’ is attached. By presenting a more in-depth study of the action sequences in which *tu vois* ‘you see’ is employed, this contribution aims to highlight the prospective potential of this interactional resource and to show that its use is consequential even when it accompanies actions that project only weakly some response from the recipient. Our findings reveal that a recurrent embodied conduct of the speaker, namely his/her gazing at the coparticipant while producing *tu vois* ‘you see,’ combined with a consistent prosodic pattern (see section “Multimodal Features of Turn-Final *Tu Vois* ‘You See”’ *infra*), enhance the interactional and prospective potential of this construction. This research examines the way in which this verbal expression is connected to the co-occurring embodied conduct of the participants – a multimodal concern that has remained practically unexplored in the previous studies on *tu vois* ‘you see’ or on other linguistic items figuring in turn-final position (in addition to the above-mentioned studies, see also Hakulinen, 2001 and Hayano, 2017, *inter alia*).

Through its concern for multimodality, this paper draws also on inquiries into the role of gaze, facial expressions, and gestures in the interactional management of turns and of conversational actions (Goodwin, 1979; Streeck et al., 2011; Deppermann and Streeck, 2018). This work builds especially on research on the interactional functions of gaze, which has been shown to be a particularly powerful resource for mobilizing recipient response or for pursuing it after lack of uptake (Kendon, 1967; Stivers and Rossano, 2010; Rossano, 2012; Auer, 2021). Of particular relevance to our endeavor here are also works that have focused on the interplay between grammar and bodily conduct. These studies have highlighted the way specific grammatical constructions are coupled with precise embodied resources for accomplishing particular actions (Keevallik, 2013, 2020; Kärkkäinen and Thompson, 2018; Pekarek Doehler, 2019; De Stefani, 2020; Stoenica, 2020; Stoenica and Pekarek Doehler, 2020).

## Data and Multimodal Features of Turn-Final *Tu Vois* ‘You See’

### Data

The database for this study consists of 28 video-recorded informal interactions between students in a Swiss university cafeteria, comprising a total of 9 h and 25 min. The recorded participants, of whom 34 are males and 33 females, agreed to sign an informed consent form for data collection and publication. The data were transcribed according to conversation analytic conventions (Jefferson, 2004; Ten Have, 2005; see Appendix). The transcription of embodied conduct followed Mondada (2018) special conventions.

In these data, 123 occurrences of turn-final *tu vois* ‘you see’ were identified. It is in this position that the construction was found to be the most recurrently used (as opposed to 17 instances in turn-initial position). Since previous research on turn-final *tu vois* ‘you see’ has documented its use merely for indexing turn completion (for an overview of the literature, see section “Background” *supra*), we aimed to better understand its interactional functioning, by investigating both the actions performed in the turns it is attached to and the participants’ co-occurring bodily conduct.

Sequential analyses revealed that *tu vois* ‘you see’ was recurrently attached to turns conveying the speaker’s emotional stance (37 instances, 30.0%), to turns performing dispreferred actions (40 occurrences, 32.5%), and to turns claiming insufficient knowledge (6 occurrences, 5.0%). In addition to this, it was also found that 95.02% of the turns belonging to these collections received a response from the recipient, making thus relevant an examination of the interactional features – in terms of praxeological and multimodal cues – that made such a sequential organization possible. The present study is based on the three collections that we have thus far sequentially and multimodally investigated. While 10 (8.1%) examples have been discarded for various technical reasons, about 15 (12.2%) of the remaining instances, which have not been systematically examined, seem to occur in informing sequences, 12 (9.8%) occurrences in storytellings and 3 (2.4%) cases in evidential vindication contexts (cf. Kendrick, 2019). Further investigation is needed to confirm these initial observations.

In what follows, we present several excerpts that illustrate the use of turn-final *tu vois* ‘you see’ in each of the above-mentioned action sequences. We show that this construction is produced with final rising intonation, and co-occurs with the speaker’s gaze at the recipient, constituting a recurrent multimodal practice through which the speaker invites a response from the recipient. This invitation for a response is distinct according to the type of actions that are performed in the turns which *tu vois* ‘you see’ is attached to: It may target, for example, a display of affiliation or a response that addresses, from the recipient’s point of view, the speaker’s claim of insufficient knowledge.

### Multimodal Features of Turn-Final *Tu Vois* ‘You See’

The French construction that we are interested in bears the same lexico-syntactic form as its English translated equivalent. More precisely, it is composed of the second person singular pronoun (*tu* ‘you’) + the verb *voir* ‘to see’ conjugated in the present indicative (*tu vois* ‘you see’), the final ‘s’ marking the verbal ending of the second person singular.

In what concerns the morpho-prosodic pattern of the construction, *tu vois* ‘you see’ is always produced as such, [ty] + [vw], that is, with two separate morpho-prosodic units (*tu* ‘you’ + *vois* ‘see’). After repeated listening to our data, we found that all occurrences of turn-final *tu vois* ‘you see,’ mobilized in the action sequences specified above (see section ‘‘Data’’), are prosodically attached to what precedes them in the turn, and are produced, with just four exceptions, with final rising intonation^3^.

The occurrence of this prosodic pattern is linked to the fact that, in French, transition relevance places (Sacks et al., 1974) are projected through mainly three prosodic features: the focal accent (‘accent nucléaire’), the melodic movement on the accentuated syllable (i.e., a rise in pitch), as well as its lengthening. In order to demonstrate the importance of the focal accent in the turn-taking process, Persson (2017) investigated its relation to overlaps in French interactions. He found that overlaps mostly occur after the accentuated syllable, which indicates that speakers orient to the focal accent as a point of possible completion for the ongoing turn constructional unit (TCU) (*ibid.:* 36). In most cases, the last or penultimate syllable carries the focal accent, which usually co-occurs with the lexico-syntactic and also the actional completion of the TCU (Persson, 2013).

The prosodic pattern that Persson (2017) identified by examining French from France is also systematically found in our Swiss-French data, including the TCUs ending with *tu vois* ‘you see’. In almost all cases, the focal accent is on the last or penultimate syllable before *tu vois* ‘you see’ and there is often a high pitch movement on the accentuated syllable. *Tu vois* ‘you see’ itself is then pronounced with lower pitch than the preceding syllable(s) but there is a rise or slight rise again on *vois* ‘see’. Taken together, these features indicate that the focal accent on the last or penultimate syllable before *tu vois* ‘you see’ and its melodic movement (a rise in pitch) already project a transition relevance place, which is actually reached after the production of the construction, reinforcing thus the relevance of a response from the recipient.

As for the third feature, the lengthening of the syllable carrying the focal accent, we observe – in some examples – a difference between Persson’s findings and ours. When a lengthening of the stressed syllable occurs, it is rather prominent. This is due to the Swiss variety of French, where the lengthening of the penultimate syllable is more frequent than in ‘standard French’ (Woehrling, 2009). According to Avanzi et al. (2015), this may not be, though, a phonological difference but a perceptual one. In their study, they conclude that it is because of the lengthening of the penultimate syllable in the Swiss variety that listeners are more likely to perceive a prominent penultimate syllable (which is not the case for Parisian French). This variety-specific feature does not influence, however, the prosodic pattern of *tu vois* ‘you see’.

Concerning the intonation contour of *tu vois* ‘you see’, we observe an equal distribution of final rising intonation when the construction is attached to turns performing a dispreferred action or to turns claiming insufficient knowledge, and a higher frequency of final rising intonation when the expression occurs at the end of turns conveying the speaker’s emotional stance.

The prosodic pattern of *tu vois* ‘you see’ is interestingly coupled with a prevalent embodied conduct: the speaker’s gaze directed toward the recipient. All turn-final instances of *tu vois* ‘you see’ in our data are combined with this gaze conduct. Kendon (1967) and, more recently, Auer (2021) have shown that the speaker’s gaze at the prospective next speaker in the last part of the ongoing turn, before a possible completion point is reached, assumes turn-transition relevance in dyadic interactions, and constitutes “the most ubiquitous next-speaker-selection technique” (Auer, 2021: 117) in multi-party interactions. This gaze conduct is systematically correlated in our data with the production of turn-final *tu vois* ‘you see’, forming a multimodal practice that acquires response-mobilizing relevance in the three praxeological environments that we have thus far identified.

These findings emerging from the data are illustrated by the excerpts in the next section (“Interactional Workings of Turn-Final *Tu Vois* ‘You See”’), their implications for the understanding of the use of *tu vois* ‘you see’ being then discussed in the last section of this paper (“Discussion and Conclusion”).

## Interactional Workings of Turn-Final *Tu Vois* ‘You See’

### *Tu Vois* ‘You See’ Attached to Turns Conveying the Speaker’s Emotional Stance

The verbal expression *tu vois* ‘you see’ is recurrently found in our data at the end of turns that display the speakers’ stance. Stance generally refers to the expression of the speakers’ position on the matter discussed, on an evaluative, epistemic, or evidential scale (Ochs and Schieffelin, 1989; Rauniomaa, 2008). Stance taking can be displayed through prosody but also through lexical choice, such as the use of evaluative terms or of different lexical items, as *I think*, for example (Kärkkäinen, 2003; Keevallik, 2003).

In our data, the issues on which speakers take a stance are often delicate and related, among other things, to their relationship with other friends, the sexual orientation of young adults, unemployment, etc. They are hence emotionally charged and their production builds on the mutual manifestation of affiliation – which refers to “the affective level of cooperation” (Stivers et al., 2011: 21) between the participants in the interaction. There are nevertheless cases in which affiliative responses, related to the stance taken by the prior speaker, although preferred (on preference organization, see section “*Tu Vois* ‘You See’ Attached to Turns Performing a Dispreferred Action” *infra*), are not produced by coparticipants. In this section, we show that the multimodal practice of producing *tu vois* ‘you see’ with a final rising intonation and with the speaker’s gaze directed to the recipient occurs in this environment. The practice is exploited in order to pursue affiliation from the interlocutor. Due to space constraints, we illustrate this by one excerpt, prototypical for our collection.

Prior to the beginning of this extract, Eliza announced to Ekta, who is seated with her back to the entrance of the cafeteria, that a mutual female friend had just arrived but gone to another room. Eliza then said that maybe this friend had not noticed them at the table, but Ekta replied the contrary, being sure that the friend only pretended to not have seen them. After stating that this friend might meet up with her boyfriend, following Eliza’s comment that she came in as if looking for someone, Ekta continues with what opens the extract:


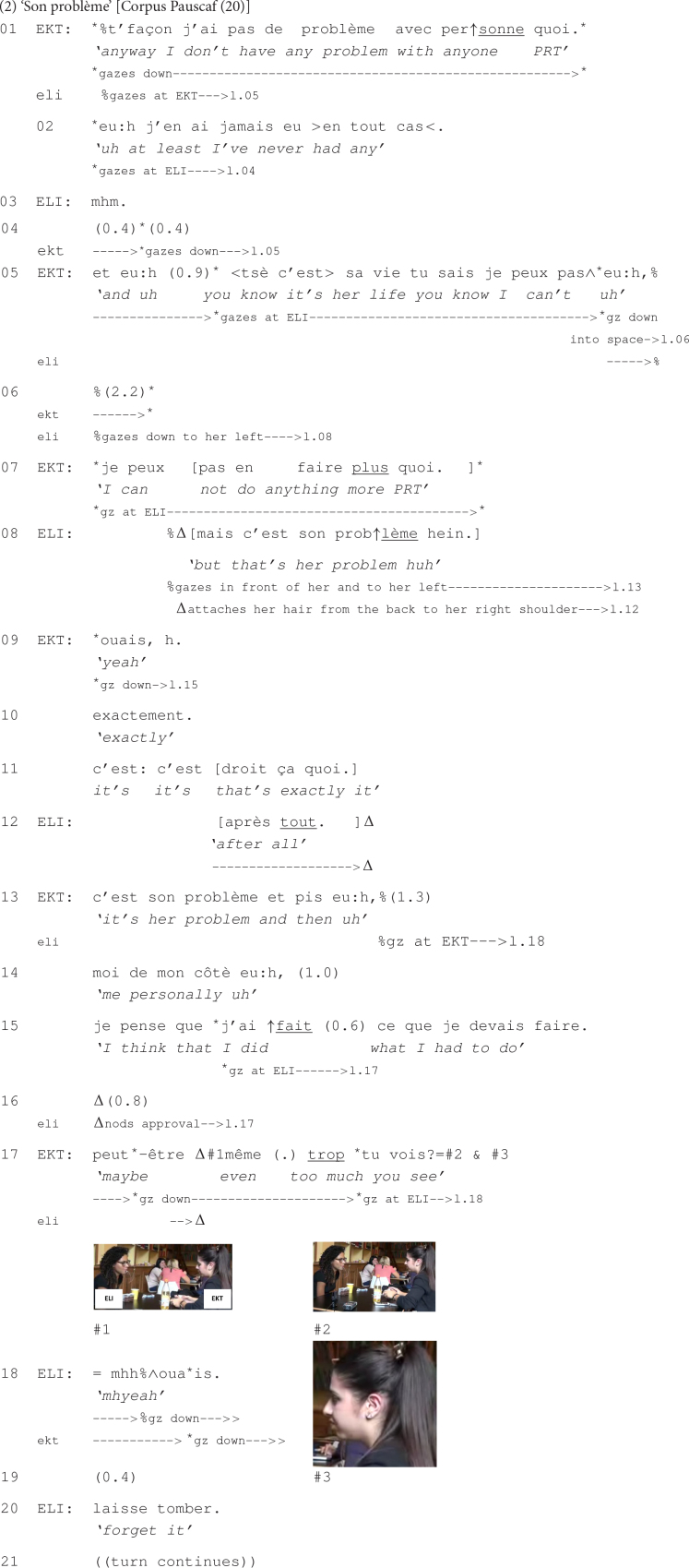


Given the exchange that precedes this extract as well as the way it begins (line 01), it is clear that Ekta’s relationship with this female friend has deteriorated. Ekta’s affirmation that she has no problem with anyone suggests thus that it’s not her fault if their relationship has become worse. While partially repeating her affirmation (line 02), Ekta directs her gaze to Eliza, inviting thus an affiliation from her part. Eliza responds only with a minimal acknowledgment token (line 03), without adding anything more during the pause that ensues despite Ekta still gazing at her.

Ekta then, hesitantly, continues her talk by stating that she cannot interfere with her friend’s life (line 05). This is inferable from her turn, which remains syntactically unfinished at the end of line 05, suggesting, together with the hesitation markers that delimit the turn, and the pause, that this is an emotional subject, which is difficult to talk about (Ochs and Schieffelin, 1989; Jefferson, 2015). This is confirmed by the extended pause that follows (line 06), especially since Eliza appears to be disengaged, by keeping silent and not gazing at Ekta. The latter still continues her turn (line 07) and, gazing again at Eliza, admits not being able to do more for saving their relationship. Her turn is overlapped by Eliza, stating that this situation is the respective friend’s problem, suggesting that Ekta should not be concerned about it (line 08). Even if, this time, Eliza produced a more elaborate response than her initial acknowledgment token (line 03), her response does not seem to be affiliating with Ekta’s stance of regret for not being able to save their friendship. Eliza’s lack of affiliation is also seen in her embodied conduct while delivering her response: She gazes in different directions and engages in self-grooming up to the end of the post-other-talk increment (Schegloff, 2016) that she subsequently adds (line 12).

Despite Eliza’s multimodal reactions conveying her disengagement from the subject discussed, Ekta still continues to talk about it (lines 13–15). Her turn is again marked with several hesitations (lines 13 and 14) and pauses (lines 14 and 15) and with Ekta’s gazing down most of the time, indicating that the subject is highly sensitive and that the words cannot be easily chosen to refer to it. Toward the end of her turn, she redirects her gaze to Eliza and states that she did what she had to do (line 15), suggesting thus that she could not be held accountable for the degradation of her relationship. Her turn reaches a transition relevance place making relevant a response from Eliza, showing some sort of appreciation of Ekta’s struggle to save her friendship. The fact that a response is expected at this point of the interaction is indicated by Ekta’s gaze at Eliza and also by the pause that follows (line 16) in the course of which Ekta keeps gazing at her interlocutor, who starts nodding approval several times.

Eliza’s minimal reaction to Ekta’s emotionally charged turn does not seem to satisfy the latter: She continues her turn, by adding an increment (line 17), through which she insists on the efforts that she has put into saving their relationship, which was too much, suggesting hence that her friend did not deserve such an implication from Ekta’s part. In pursuit of an affiliative response from Eliza, Ekta recompletes her turn by using, this time, the *tu vois* ‘you see’ construction delivered with a rising intonation and combined with Ekta’s gaze directed to Eliza. Additionally, Ekta displays a grimace of dislike (see #2 at line 17), through which she facially suggests that she did more than her friend deserved (see Kaukomaa et al., 2015 on facial expression and the establishment of a stance that is withheld in the talk). At the same time, by deploying this facial expression while carefully gazing at Eliza, Ekta seems to be inviting the latter to share her stance (Kaukomaa et al., 2013). The deployment of all these multimodal resources appears to be eventually successful as Eliza reacts immediately (line 18), first by aligning with what Ekta has said and then, after a short pause (line 19), by providing an affiliative response (lines 20–21), not entirely reproduced here, in which Eliza is giving Ekta some advice on how to cope with this troublesome situation.

This extract has thus illustrated the use of turn-final *tu vois* ‘you see’, produced with final rising intonation and with the speaker’s gazing at the recipient, for successfully mobilizing an affiliative response from the coparticipant in an extended sequence that offers several opportunities for the interlocutor to display affiliation, but these have not been taken.

### *Tu Vois* ‘You See’ Attached to Turns Performing a Dispreferred Action

Preference organization refers to “practices through which certain interactional outcomes are promoted or favored vis-à-vis other outcomes” (Clayman, 2002: 230). An important function of preference organization is “allowing interactants to avoid interpersonal conflict and promote solidarity” (Robinson, 2020b). Responsive actions that are cooperative or affiliative constitute preferred responses (Sidnell, 2010). In contrast, responsive actions that do not satisfy the first action’s goal are uncooperative or disaffiliative and constitute dispreferred responses (for a review of preference organization, see Clayman, 2002; Schegloff, 2007a; Pillet-Shore, 2017).

Most social actions prefer responses that are aligning with their objectives or goals. According to Robinson (2020a): “*For a majority of social actions, such alignment is achieved with answer types that can be glossed as “agreement”* (including affirmation, acceptance, confirmation, acquiescence, etc.) (*ibid*.: 426, original emphasis). This idea points to the existence of a general preference for agreement in conversation (Pomerantz, 1984; Sacks, 1987; Stivers et al., 2009). In this section, we present three prototypical excerpts for the types of dispreferred actions found in our data: disagreement (ex. 2), refusal (ex. 3), and display of a divergent stance (ex. 4). We show that, in these dispreferred action sequences, speakers make use of the *tu vois* ‘you see’ construction, while simultaneously gazing at the interlocutors, in order to mobilize an aligning response from their recipients.

The following excerpt is taken from a conversation between Joanne, Amanda, and Nathan, who talk about how to draw a box plot for 27 observations, an exercise that they need to do for their course in descriptive statistics. Prior to the beginning of this fragment, Nathan complained that he was unable to do the exercise. Joanne then volunteered to explain the procedures for the completion of the task, but she was interrupted by Nathan, willing to demonstrate his own understanding of what she had thus far explained. Joanne reacts with what opens the extract:


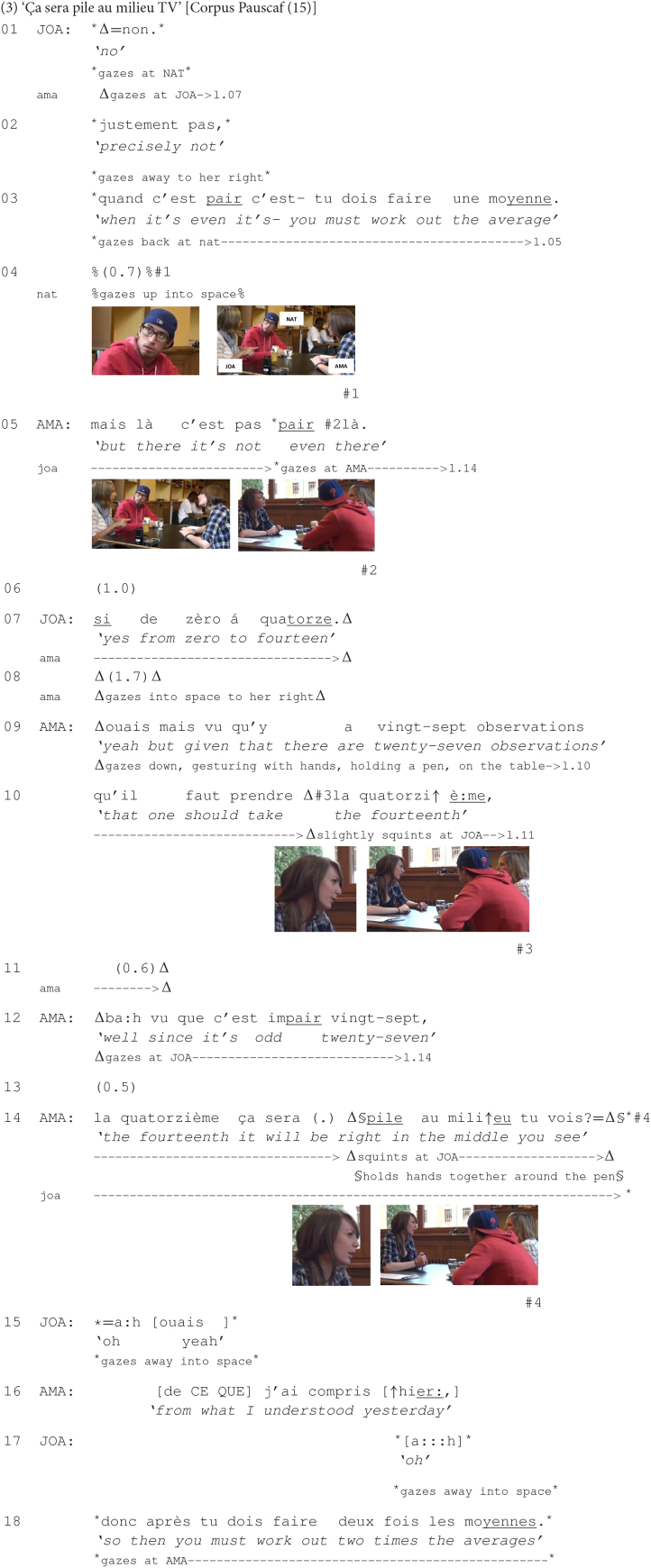


The excerpt begins with Joanne who openly disagrees with Nathan’s understanding (lines 01–02). She then provides an account meant to support her disagreement: When dealing with an even number, one must work out the average (line 03). After a pause (line 04), during which Nathan does not react verbally to Joanne’s turn but instead gazes up into space (see #1), ‘doing thinking’ (Goodwin and Goodwin, 1986), Amanda, who has thus far monitored the exchange, seizes the opportunity to come in (line 05), in response to Joanne’s turn. She plainly disagrees with Joanne’s prior account, by stating that the number that they are dealing with in their exercise is not even.

Amanda’s turn engenders a change in Joanne’s embodied conduct (see #2) as she shifts her gaze from Nathan to Amanda, who becomes thus Joanne’s focus of attention (Goodwin, 1981) until the end of the excerpt. After a subsequent pause (line 06), the latter takes the turn and openly disagrees with her colleague’s turn. Joanne persists that they are dealing with an even number, namely from zero to fourteen (line 07).

In response to this, Amanda first takes a long moment of reflection, shifting her gaze from her interlocutor into space, to her right (line 08). She then launches an explanation based on several premises designed to substantiate her prior disagreement (line 05) and thus to make Joanne agree with her. The first two premises are formally introduced by the conjunctive phrase ‘vu que’ *given that* (lines 09 and 12) and by its elliptical form ‘que’ *that* (line 10). Their production is bodily emphasized by Amanda’s “narrowed eyes” (cf. Kendon, 1967: 32) (see #3 at line 10) and by her manipulating and pointing her writing tool into the tabletop, indexing that what is being said at that very moment is highly relevant for the understanding of why Joanne’s reasoning is wrong. After producing these two arguments, Amanda pauses (l. 11) and keeps squinting at Joanne, offering the latter an opportunity to react.

As no reaction is forthcoming from Joanne, Amanda continues her turn (l. 12) and provides a third premise, further substantiating her initial disagreement. While still gazing at Joanne, Amanda pauses again, offering hence the latter, in a stepwise fashion, a second (sequential) opportunity to react, which is again not seized (l. 13). Amanda then formulates the conclusion that ensues from the three prior premises and exposes the main argument meant to prove that Joanne’s reasoning was wrong (l. 14). In pursuit of an agreement from Joanne, Amanda marks the end of her turn by using the *tu vois* ‘you see’ construction, delivered with a final rising intonation. The production of this construction is additionally coupled with Amanda’s squinting at Joanne (see #4 at line 14), emphasizing that a response from the latter is relevant at that point of the interaction. This multimodal conduct in this sequential position allows thus Amanda to mobilize a response from her coparticipant, which has been thus far unsuccessfully pursued.

This multimodal conduct appears to trigger the expected response as Joanne finally reacts (l. 15, 17–18), by agreeing with Amanda: The use of the change-of-state token ‘a:h’ *oh* (Heritage, 1984) (l. 15, 17) indexes here her figuring out how to correctly solve the exercise. The use of the acknowledgment token ‘ouais’ *yeah* (l. 15), as well as her indication of how to correctly do the exercise (l. 18) show Joanne’s agreeing with Amanda, suggesting thus indirectly that her initial reasoning was wrong.

Speakers often orient to the dispreferred nature of their actions. This is especially reflected in the design of their turns, which are marked by hesitations, cut-offs, and, according to the type of actions that they are responsive to, apologies or expressions of gratitude (Hutchby and Wooffitt, 2008; Pillet-Shore, 2017). It has been argued that such delay practices contribute to both projecting and easing the reception of dispreferred responses (Maynard, 2003; Robinson, 2020b). Therefore, refusals, for example, are routinely mitigated by excuses, which are designed so as to be accepted by the coparticipants.

This is what happens in the next excerpt, which corresponds to the beginning of a new interactional sequence, following talk about the whereabouts of a common friend that spent her last weekend in France, with her family. At line 01, Camille informs Cédric that she and her boyfriend would very much like to go to Lille, the city where Cédric comes from. She thus indirectly self-invites herself and her boyfriend to Cédric’s family home. In the lines that have been omitted due to space limitations, the latter reacted by first stating that the next two weekends his parents were not home, suggesting hence that the circumstances were suitable for such a visit. He then announced that he would not be available the very next weekend because a friend of his was celebrating her birthday and had planned some activities, as stated in the following:


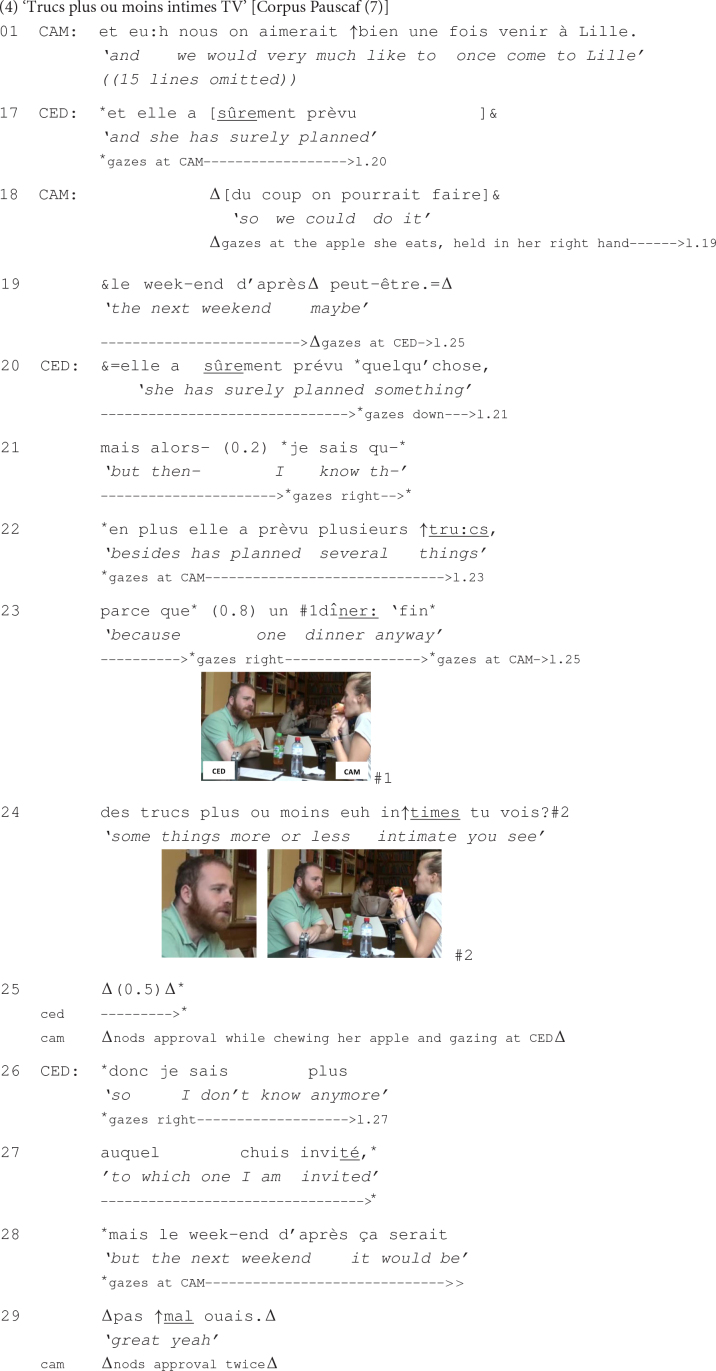


At line 17, Cédric begins to formulate an excuse for why he could not respond favorably to Camille’s self-invitation to his home. The production of the excuse is clearly indicative of his orientation to the dispreferred nature of his refusal. The excuse consists in the listing of several activities that his friend has planned for her birthday and in which he is to participate the respective weekend. Camille promptly orients to Cédric’s unavailability, as she comes in at lines 18 and 19, overlapping Cédric’s beginning of the turn, and proposes to him, in that case, to visit his parents’ place the following weekend.

Although he carefully gazes at Camille while the latter produces her proposal, Cédric does not immediately react to her turn but continues instead his excuse (lines 20–24), meant to account for his unavailability that very weekend. Cédric’s turn comprises several features that are particularly indicative of its unplanned, emergent occurrence: It is produced with several self-initiated self-repairs (lines 21 and 23) and is delivered in terms that remain quite vague, such as ‘quelque chose’ *something* (line 20) or ‘fin des trucs plus ou moins euh intimes’ *anyway some things more or less uh intimate* (lines 23–24). These characteristics emphasize that Cédric’s response to Camille’s self-invitation is produced ‘on the fly.’

At the same time, Cédric’s repetition, at line 20, of the beginning of his turn (line 17) that has been overlapped by Camille suggests that he orients to the implicative nature of his action, to which his interlocutor is hence expected to respond (Schegloff, 1987). This can be also seen in the fact that Cédric insists on providing this excuse, despite Camille’s revised self-invitation to a time when he is potentially available (lines 18–19). Cédric appears to make sure that his coparticipant accepts his excuse: He exploits the construction ‘tu vois’ *you see*, produced with a final rising intonation, at the end of his turn (line 24) as a resource to mobilize a response from Camille. Moreover, after having gazed in several different directions (see lines 20, 21, and 23), toward the end of his excuse and while producing this construction, Cédric gazes at Camille, making thus relevant that a response from her is expected at that point of the interaction. Camille responds to this by nodding approval (line 25) and gazing at Cédric, while chewing her apple, which she started eating before the beginning of the extract.

Camille’s response, though minimal, is monitored by Cédric’s gazing at her and appears to be treated by the latter as sufficient indication of her accepting his excuse, especially since she continues chewing her mouthful of apple that she finally swallows only after the end of the quoted excerpt. It is hence only now, after having received the acceptance of his excuse, that Cédric orients to Camille’s proposal of visiting his parents’ place the following weekend, which he assesses positively (lines 28–29).

Excerpts (3) and (4) have illustrated a multimodal practice – consisting of turn-final *tu vois* ‘you see’ delivered with final rising intonation and combined with the speaker’s gaze at the recipient – that speakers recurrently exploit in our data in order to invite an aligning response (agreement in ex. 3; acceptance in ex. 4). These findings are additionally borne out by the following excerpt in which the coparticipant’s response is missing (lines 10–13), despite its being targeted by the speaker using the *tu vois* ‘you see’ construction at the end of his turn. The noticeable absence of the recipient’s response leads the speaker to engage in further interactional work that is designed to repair this problem of uptake.

In this next excerpt, Ekti and Joanna are planning to organize a trip to Europa-Park, in Germany, with a group of friends. Prior to the beginning of this extract, they weighed whether to go by car, with one of their friends named Carla as a driver, or to take the bus. Eventually, they decided that the train would be the best option, allowing them to travel with the rest of their friends, as a group. Ekti then continues with what opens the excerpt, namely with her stating that Carla does not have anyway the necessary experience to drive to Germany.


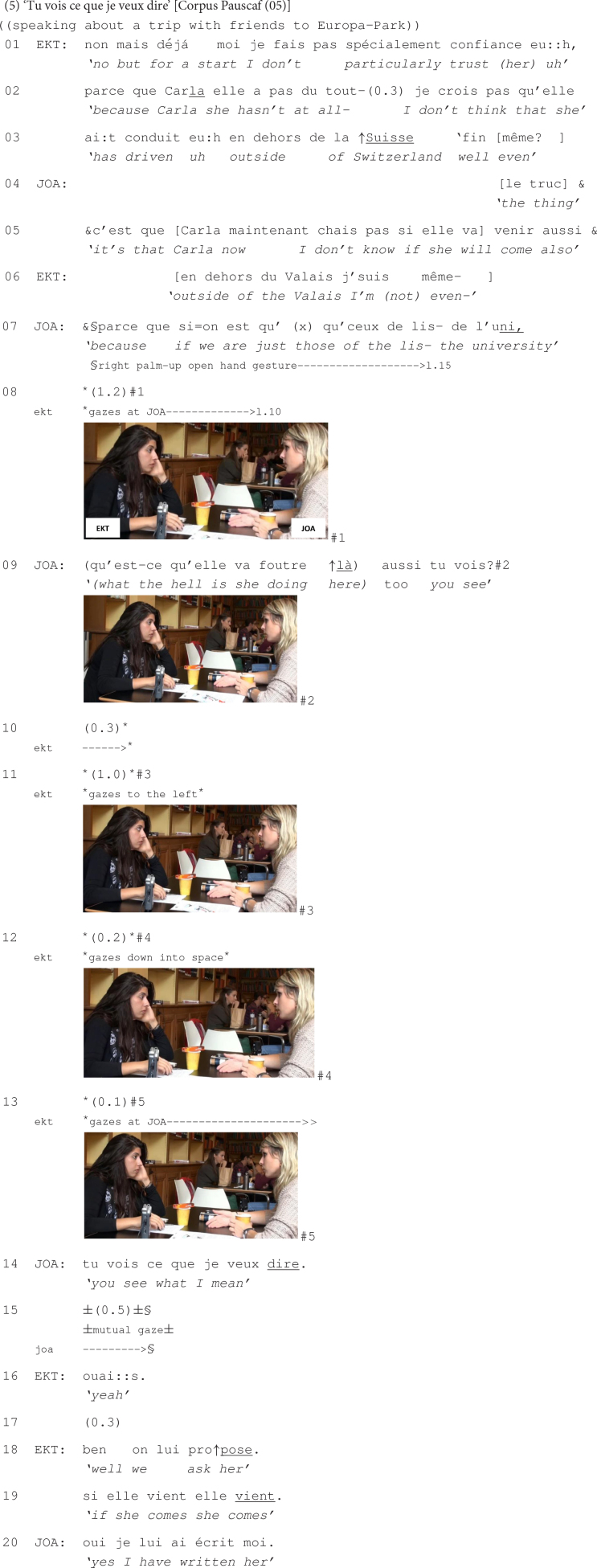


At lines 01 to 03 and 06, Ekti expresses her mistrust of Carla’s skills to drive to a foreign country. While accounting for her apprehensive stance, Ekti gets interrupted by Joanna (lines 04–05). This dispreferred way of taking the turn is additionally coupled with Joanna’s disaffiliative stance: She downgrades the likeliness of Carla actually joining the group, dismissing thus the grounds for Ekti’s concerns. She then provides an account, in the form of a syntactically incomplete if-clause, for why Carla may be unwilling to join them (line 07). Moreover, when producing this account, Joanna stresses the word ‘l’uni’ (the abbreviation of *university*) and deploys a palm-up open hand gesture emphasizing the obviousness of her argument (cf. Kendon, 2004: 266) on British and American English and Italian data meant to make Ekti agree that their friend may not actually come. During the extended pause that follows (line 08), Joanna keeps gazing at Ekti while maintaining her palm-up open hand gesture directed to her coparticipant (see #1 at line 08), inviting thus the latter to react (cf. Streeck, 2009 research on gesture as embodied communicative action in multiple languages: French, (Turkish-)German, Thai, Japanese, and Ilokano).

As no reaction is forthcoming from Ekti, Joanna continues her turn (line 09) by affirming that Carla’s place is not in this group. This membership categorization (Sacks, 1992; Schegloff, 2007b), emphasized also by the cursing expression that Joanna uses, is exploited here as another argument meant to convince Ekti that there is little chance for Carla to come along, suggesting once more that Ekti should not be worried about the idea of traveling abroad by Carla’s car. In a second attempt to make Ekti react, Joanna mobilizes the *tu vois* ‘you see’ construction with final rising intonation at the end of her turn, inviting the former to agree that Carla may not come after all because her place is not among fellow students. The use of this verbal construction in the pursuit of a response from the recipient is bodily coupled with Joanna’s fixed gaze on Ekti and with her maintained palm-up open hand gesture directed to the latter (see #2 at line 09).

Despite these verbal and embodied resources deployed by Joanna, her pursuit of a response from Ekti is not successful. Ekti first gazes to the left (see #3, line 11), probably distracted by loud laughter that is heard at that moment in the cafeteria, and then she gazes down into space (see #4 at line 12). Note that all this time, Ekti’s embodied conduct is carefully monitored by Joanna who keeps gazing at her, while still maintaining her palm-up open hand gesture directed to the latter, indexing thus bodily that a response from Ekti is relevant at that point of the interaction.

Orienting to Ekti’s noticeable absence of response, Joanna produces a question (line 14), designed to check whether Ekti has properly attended to her talk thus far. The fact that Joanna launches this enquiry in this sequential position suggests that she treats Ekti’s lack of response as a result of her not having paid enough attention to Joanna’s prior talk. The question is thus meant to solve the interactional problem that Joanna orients to as having caused the absence of a response from Ekti at a moment where it has been made especially relevant (on sequential repair, see Houtkoop-Steenstra and Antaki, 1997).

Interestingly, it is only after producing the answer to this question, by Ekti’s confirming her attendance (line 16), that the latter provides the response that has been previously expected by Joanna (lines 18–19). Still, this response appears to be disaligning with Joanna’s prior talk. Ekti’s response, in this sequential position, indicates that her lack of reaction has not been due to her not attending to Joanna’s talk but rather to her reluctance to agree with the latter’s arguments concerning Carla’s potential absence from their excursion. This is additionally seen in the design of her response as she proposes to nevertheless invite Carla to their trip. The sequence is then closed with Joanna’s agreeing with this and announcing that she has already written to Carla.

This excerpt has illustrated that when the response that is called for by the multimodal use of the *tu vois* ‘you see’ construction is noticeably absent, the speaker may engage in further interactional work so as to remedy this problem of uptake.

### *Tu Vois* ‘You See’ Attached to Turns Claiming Insufficient Knowledge

A third environment in which speakers of our data deploy the multimodal practice involving *tu vois* ‘you see’ is at the end of turns containing the expression ‘je (ne) sais pas’ *I don’t know* or in combination with other linguistic items (i.e., ‘je sais pas trop’ *I don’t know much*; ‘je sais plus’ *I don’t know anymore*). These turns occur as part of responsive or initiating actions. When occurring as responsive actions, these turns are used to display responsiveness without giving an explicit answer (Beach and Metzger, 1997). In this case, the turns are composed only of ‘je (ne) sais pas’ *I don’t know* or the above-mentioned variants to which the *tu vois* ‘you see’ construction is attached. Additionally, these turns may index approximation (Weatherall, 2011) or project a disaligning answer, in which case the ‘je (ne) sais pas’ *I don’t know* expression fulfills a hedging function (Keevallik, 2011; Pekarek Doehler, 2016; for an overview of epistemic hedges, see Lindström et al., 2016). The two types of responsive turns, ending with *tu vois* ‘you see’ delivered with a final rising intonation, coupled with the speaker’s gaze directed at the recipient, systematically receive – just as in the excerpts belonging to the other collections (see section “*Tu Vois* ‘You See’ Attached to Turns Conveying the Speaker’s Emotional Stance” and section “*Tu Vois* ‘You See’ Attached to Turns Performing a Dispreferred Action” *supra*) – a response from the coparticipant, acknowledging thus the prior response.

When these turns occur in initiating actions, the ‘je (ne) sais pas’ *I don’t know* expression is followed by a complement clause (see ex. 6 *infra*) to which the *tu vois* ‘you see’ construction is attached. These turns convey the speaker’s relatively unknowing stance (Robinson, 2020b) or uncertainty (Beach and Metzger, 1997) about the matters at hand. In this section, we argue that, in the described environment, the use of the multimodal practice entailing *tu vois* ‘you see’ contributes to mobilizing a response addressing the speaker’s claim of insufficient knowledge from the interlocutor’s own perspective. The response that is hence targeted is not just an acknowledging response, it is a reaction revealing the recipient’s point of view or experience about the matter discussed. Due to space limitations, we illustrate this phenomenon with one excerpt.

Before the beginning of the extract, Alexeï and Eddy talked about the first name of a mutual acquaintance whose pronunciation would suppose a nasalization of the last syllable but which professors, according to Alexeï, often do not produce when calling his name. The sequence is closed and a pause occurs in the course of which both participants gaze down to the sheets of paper in front of them. Alexeï then reopens the sequence with what follows:


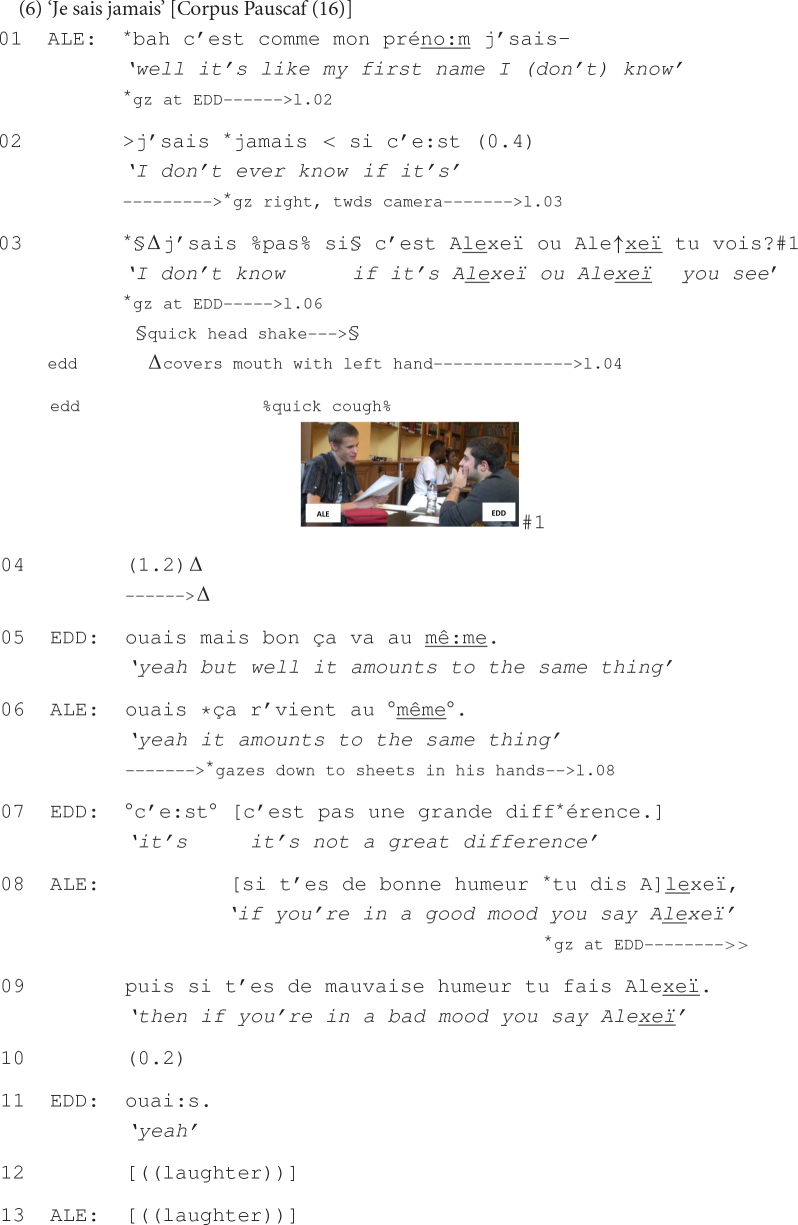


At lines 01 to 03, Alexeï expands the previously closed sequence on the mispronunciation of their acquaintance’s first name, by referring to his own given name. In this sense, his use of ‘c’est comme mon prénom’ *it’s like my first name* (line 01) from the very beginning of his turn signals that a similar pronunciation error to the one previously talked about may concern his own forename. Then, after a cut-off (line 01), two self-initiated self-repairs (line 02 and 03), and a pause (line 03), Alexeï states that he does not know if his name should be pronounced with a stress on the second (Alexeï) or on the last syllables (Alexeï) (line 03).

In this sequential position, Alexeï’s claim of insufficient knowledge, also bodily enacted through a quick head shake (see line 03) conveys his uncertainty (Beach and Metzger, 1997) regarding the correct pronunciation of his first name, according to the two possibilities. This is also suggested by his use of ‘je sais jamais si c’est’ *I don’t ever know if it’s* (line 02), which indicates that this matter has always been a dilemma for him. Alexeï marks the end of his turn by employing *tu vois* ‘you see’, produced with final rising intonation, while carefully gazing at Eddy (see #1 at line 03). This verbal construction seems to be multimodally exploited at this point of the interaction in order to mobilize a response from Eddy that possibly addresses Alexeï’s dilemma. Attached to Alexeï’s claim of insufficient knowledge, this construction appears to be doing more than just inviting, for instance, an acknowledging reaction: It sets the relevance for a response that would target the preceding claim, but from the recipient’s own perspective.

The fact that Alexeï is expecting a response from his interlocutor is also confirmed by his gaze at Eddy, maintained throughout the extended pause that follows (line 04). Alexeï’s prolonged gaze at Eddy contributes to reinforcing the sequential implicativeness of his prior turn, emphasizing thus the relevance of a response from his interlocutor (on the role of prolonged gaze in pursuit of a response, see Stivers and Rossano, 2010). This is oriented to by Eddy, who takes the turn and provides more than just an acknowledging response: He states that the changing accentuation does not make a big difference (line 05). By affirming that the two pronunciations amount to the same thing, Eddy suggests that, from his point of view, the difference between the two pronunciations of Alexeï’s name is less consequential than the difference between a nasalized and a non-nasalized sound. This is repeated once again by Eddy (line 06), after receipt of Alexeï’s agreement (line 05), and oriented to by the latter with a joke (lines 08–09), suggesting that he should not indeed take this dilemma seriously. The joint laughter that ensues from the joke marks the end of the sequence.

This last excerpt has shown that in sequences involving the speaker’s claim of insufficient knowledge the use of turn-final *tu vois* ‘you see’, with final rising intonation and with the speaker’s gaze at the recipient, contributes to eliciting more than just an acknowledging response: It invites the interlocutor’s point of view about the matter discussed.

## Discussion and Conclusion

This paper has documented the use of turn-final *tu vois* ‘you see’ in video-recorded ordinary conversations in French. The sequential and praxeological investigation of the data has revealed that the *tu vois* ‘you see’ construction is recurrently attached to turns conveying the speaker’s emotional stance (see section “*Tu Vois* ‘You See’ Attached to Turns Conveying the Speaker’s Emotional Stance” *supra*), to turns performing a dispreferred action, like disagreeing (see section “*Tu Vois* ‘You See’ Attached to Turns Performing a Dispreferred Action” *supra*), and to turns claiming insufficient knowledge (see section “*Tu Vois* ‘You See’ Attached to Turns Claiming Insufficient Knowledge” *supra*). In each of these distinct praxeological environments, it has been found that speakers exploit *tu vois* ‘you see’ in order to mobilize a response from their interlocutors. The analyses have shown that the response that is invited through the use of this construction differs from one action sequence to another: an affiliative response (see ex. 2), an aligning response (see ex. 3, 4, and 5), and a response addressing the prior speaker’s claim of insufficient knowledge from the recipient’s own perspective (see ex. 6).

The response-mobilizing potential of this construction has been evidenced to rely on an intricate layering of semiotic resources (Goodwin, 1979, 1981) that are finely tuned. Linguistically, the *tu vois* ‘you see’ expression represents, through the second person pronoun, a form of addressing the interlocutor. Together with the verb, ‘voir’ *to see*, it projects, structurally speaking, a yes/no type of response and thus at least a minimal response. The form of this response depends on the conversational action performed by the turn to which *tu vois* ‘you see’ is attached (see in this sense section “*Tu Vois* ‘You See’ Attached to Turns Claiming Insufficient Knowledge”). Prosodically, the production of the construction with a final rising intonation, similar to the final intonations of questions in French (Déprez et al., 2013), enhances its potential of mobilizing a response from the recipient. Additionally, *tu vois* ‘you see’ is preceded by the accentuated syllable that alerts the interlocutor to an upcoming transition relevance place, which is actually reached after the production of the construction, reinforcing thus the relevance of a response from the recipient (see section “Multimodal Features of Turn-Final *Tu Vois* ‘You See”’). Bodily, the expression is systematically coupled with the speaker’s gaze directed to the recipient, a gaze pattern that signals turn completion and allocation in dyadic interaction (Kendon, 1967) and constitutes a powerful resource to select a next speaker in multi-party conversation (Auer, 2021).

These dynamically deployed semiotic resources form a multimodal practice that is recurrently exploited by speakers in our data for mobilizing a response from their interlocutors, even in praxeological environments that only moderately project actions from the coparticipant(s) (see, for example, section “*Tu Vois* ‘You See’ Attached to Turns Claiming Insufficient Knowledge”). It is also this multimodal practice that enables speakers to eventually obtain a relevant response after several unsuccessful pursuits of such a response from their interlocutors (see ex. 2 and 3). The use of this multimodal practice provides recipients with a sequential opportunity for displaying co-participation (Goodwin, 1979, 1981; Hayashi, 2003; Fox, 2007) by providing a response when it becomes interactionally relevant. Recipients, in turn, are seen to orient to this conditional relevance, as evidenced by the fact that they provide the relevant next, being held accountable when such appropriate response is noticeably absent (see ex. 5).

The investigation of the praxeological environments in which turn-final *tu vois* ‘you see’ is used points also to the fact that the sematic interpretation of this construction depends on the action sequence it occurs in and on the type of response that it targets. When the construction is employed for inviting an aligning response (see section “*Tu Vois* ‘You See’ Attached to Turns Performing a Dispreferred Action” *supra*), it seems to appeal to the interlocutor’s intellectual comprehension of the talk that it is attached to, confirming thus Andersen (1997: 196–197). When the construction is used for inviting an affiliative response (see section “*Tu Vois* ‘You See’ Attached to Turns Conveying the Speaker’s Emotional Stance” *supra*) or a response that would target the speaker’s claim of insufficient knowledge (see section “*Tu Vois* ‘You See’ Attached to Turns Claiming Insufficient Knowledge” *supra*), it appears to convey “an invitation not to seeing but to the experiential sharing […] in an empathic way” (Détrie, 2010: 765)^4^. The examination of the interactional functioning of turn-final *tu vois* ‘you see’ contributes thus to highlighting that the semantic interpretation of this construction, in the action sequences that have been investigated, is intricate and departs from the basic meaning of visual perception. This is in line with recent discussions on the conversational use of *you see* in Estonian (Amon and Keevallik, 2020).

These findings suggest that the multimodal practice involving turn-final *tu vois* ‘you see’ builds on a complex layering of semiotic resources and is intimately intertwined with the sequential organization of the conversational actions and the co-occurring interactional contingencies. These results could constitute the basis for a future research on turn-final *tu vois* ‘you see’ in other action sequences, such as informings or storytellings (which we have thus far only preliminarily identified, see section “Data”), as well as in other types of interactional settings. In the same vein, further research could look more thoroughly into the way in which the deployment of certain gestures, like the palm-up open hand gesture (see ex. 5), may contribute to enhancing the response-mobilizing potential of the *tu vois* ‘you see’ construction. Finally, the present study could be complemented by a future research on the multimodal use of this verbal expression across several languages so as to verify if the identified multimodal practice represents a phenomenon that participants orient to cross-linguistically and cross-culturally.

## Data Availability Statement

The original contributions presented in the study are included in the article/supplementary material, further inquiries can be directed to the corresponding author/s.

## Ethics Statement

Ethical review and approval was not required for the study on human participants in accordance with the local legislation and institutional requirements. The patients/participants provided their written informed consent to participate in this study. Written informed consent was obtained from the individual(s) for the publication of any potentially identifiable images or data included in this article.

## Author Contributions

Both authors have equally contributed to the design of the work and approved it for publication.

## Conflict of Interest

The authors declare that the research was conducted in the absence of any commercial or financial relationships that could be construed as a potential conflict of interest.

## Publisher’s Note

All claims expressed in this article are solely those of the authors and do not necessarily represent those of their affiliated organizations, or those of the publisher, the editors and the reviewers. Any product that may be evaluated in this article, or claim that may be made by its manufacturer, is not guaranteed or endorsed by the publisher.

## Footnotes

1 As a reminder, a discourse marker is defined “as a phonologically short item that is not syntactically connected to the rest of the clause (i.e., is parenthetical), and has little or no referential meaning but serves pragmatic or procedural purposes” (Brinton, 2008: 1). Just as it is the case with the verbal expression that we are interested in, the category of discourse markers has been called many names, revealing thus the heterogeneous nature of the linguistic items that it comprises. We can thus find in French labels such as: *mots du discours* (Ducrot, 1980), *connecteurs* (Roulet et al., 1985), *opérateurs* (Anscombre and Ducrot, 1983; Anscombre et al., 2018), *marqueurs de structuration de la conversation* (Auchlin, 1981), *ponctuants* (Vincent, 1993), *petits mots de l’oral* (Bruxelles and Traverso, 2001), and *marqueurs discursifs* (Dostie and Pusch, 2007). In English, we can find the following non-exhaustive denominations: *discourse markers* (Schiffrin, 1987; Mosegaard Hansen, 1998; Auer and Maschler, 2016; Mazeland, 2016), *particles* (Heritage and Sorjonen, 2018), and, according to Fraser (1999), who cites the relevant references: *cue phrases*, *discourse connectives*, *discourse signaling devices*, *phatic connectives*, *pragmatic markers*, *pragmatic operators*, *pragmatic particles*, and *sentence connectives*.

2 The difference between *pragmaticalization* and *grammaticalization* (Hopper and Traugott, 2003) is much debated in the literature. Without aiming to contribute to the debate, which goes beyond the scope of this paper, we refer here to Erman and Kotsinas (1993) view on these two phenomena, as it is their perspective that has been adopted by several authors, like Bolly (2010, 2012) and Dostie (2004), who examined the French verbal expressions that we are interested in.

3 Following preliminary analyses, this prosodic pattern has appeared to be consistent even in the cases that we have not included in our collections.

4 Our translation of: “[…] une invitation non au voir, mais au copartage expérientiel […], sur un mode emphatique.” (Détrie, 2010: 765).

## Appendix

***Transcription conventions for verbal conduct.***

**Table:**

[	start of overlap
]	end of overlap
=	latching (no pause, no overlap)
(0.7)	measured pause in seconds and tenths of seconds
wo-	truncated word
wo:rd	syllable lengthening
?	final rising intonation
.	final falling intonation
,	continuing intonation
Word	accentuation
°word°	softer than surrounding speech
WORD	louder than surrounding speech
↑word	marked rise in pitch (refers to the next syllable)
∧	liaison
.h	in-breath
h.	out-breath
((laughter))	transcriber’s comment

***Transcription conventions for embodied conduct.***

For more details, see Mondada (2018).

**Table:**

* *	Indicates start and end of gaze or of gaze and other embodied conduct (e.g., nodding) of speaker A.
§§	Indicates start and end of another relevant embodied conduct (e.g., pointing) of speaker A.
? ?	Indicates start and end of an embodied conduct of speaker B.
% %	Indicates start and end of an embodied conduct of speaker B.
±±	Indicates start and end of an embodied conduct mutually displayed by two participants (e.g., gaze at each other).
*—->l.12	Continuation of the described embodied conduct until line 12 of transcript.
——->*	End of the described embodied conduct.
*—–>>	Continuation of the described embodied conduct until end of excerpt.
——>>*	Described embodied conduct has been going on since the beginning of the excerpt
